# Evaluation of healing following frenectomy

**DOI:** 10.6026/973206300171138

**Published:** 2021-12-31

**Authors:** Nurul Afiqah Amani Binti Zaaba, Arvina Rajasekar, Shantha Sundari KK

**Affiliations:** 1 Saveetha Dental College and Hospitals, Saveetha Institute of Medical and Technical Sciences, Saveetha University, Chennai - 600 077, India

**Keywords:** Conventional scalpel, High frenal attachment, Healing, Laser technique

## Abstract

Aberrant frenum attachment would cause plaque accumulation and malalignment of teeth. It can be managed by frenotomy or frenectomy methods, through a conventional surgical technique or laser technique. Therefore, it is of interest to compare frenectomy healing surgical and laser techniques. Data from 51 outpatients and post-operative healing of frenectomy was assessed by Landry’s healing score index using 3 weeks postoperative photographs followed by statistical analysis. Based on the healing score index, the laser technique showed better outcomes than the surgical technique. Moreover, the association between the management of high frenal attachment and the healing score index was found to be statistically significant.

## Background:

A frenum is also known as a frenulum, which is a small band or fold of mucosal membrane and connective tissue fibers seen in the oral cavity, which is surrounded by muscle fibers [1]. They are connected to the alveolar mucosa and to the underlying periosteum from the cheeks and lips [1,2]. It commonly can be seen around maxillary and mandibular incisors as well as on canine and premolar areas [3]. In general, frenal attachment can be classified into mucosal, gingival, papillary, and papillary penetrating depending upon the attachment of muscle fibers. However, papillary and papillary penetrating frenum are considered to be pathological when seen clinically [1], as it can compromise the normal functions with restricted movement as well as aesthetic appearances. Basically, high frenum attachment is caused by muscle pull that leads to the opening of the gingival sulcus, which in turn results in plaque accumulation and malalignment of teeth [4]. In addition to this, it could lead to loss of papilla, gingival recession, and also midline diastema [1]. There are various methods that can be used to check for high frenal attachment. The most common method used for diagnosis is the blanch test [5]. It is normally done through the application of forces on the frenum by pulling it away from the mucosa. Any clinical movement seen on the papillary tip or blanching [3], will help to detect the presence of aberrant frenum attachment. These abnormalities can be easily managed by different treatment modalities, which are frenectomy or frenotomy methods, depending upon the size and location of the frenum. This should be accomplished by locating and assessing the proximity of different anatomical structures in relation to the planned operative site [6]. Treatments should focus on minimizing tissue trauma in order to preserve the tooth-gingival contour in terms of both aesthetics and functionality [7]. Thus, patients should have considerable knowledge of the influence of various risk factors and efficacy [8] of each technique. During a frenectomy, the frenulum, as well as its attachment to the underlying alveolar process is entirely removed during the treatment. Frenotomy is done using simple excision to release frenum from the apex of its insertion to its base and down to the alveolar process [2]. Although tissue excision is different in both methods, the original architecture, healthy dentition, and functionality of the periodontal tissues should be maintained [9,10]. A frenectomy can be easily done by surgical or laser technique. The surgical technique is the earliest technique introduced which is known as a conventional scalpel technique or “classical frenectomy" by Archer and Kruger. However, it can cause labial tissue scarring, which may be unaesthetic. Thus, to overcome this limitation, Miller advocated a surgical technique combining frenectomy with a laterally displaced flap in 1985, which is aesthetically acceptable and results in wound healing by primary intention [11]. Over the years, the laser technique is introduced to treat aberrant frenum as an alternative method with better results than surgical technique. The majority of studies reported that the laser technique was done with less bleeding and less pain experienced by the patients along with less usage of analgesics during the procedures [1,3,4]. On top of that, no scar formations were seen on the operated area [12], with no tissue destruction [13]. This is due to the capacity of the periodontal tissues to regenerate [14]. Although wound healing is delayed in the laser approach, better clinical and healing outcomes could be seen postoperative with diode laser in comparison to conventional scalpel technique [1,[4]. Thus, dental practitioners are using laser techniques to treat aberrant frenum than surgical methods. Therefore, it is of interest to evaluate frenectomy healing using surgical and laser techniques.

## Materials & Methods:

A retrospective study was designed to compare frenectomy healing using surgical and laser techniques. Between June 2019 and March 2020, the study used case records from patients at a private institution. The Institutional Research Committee granted permission to use the data for study and analysis in advance. SDC/SIHES/2020/DIASDATA/0619-0320 is the ethical approval number. A total of 51 frenectomy patients were enrolled in this study. The patients were divided into two groups based on frenectomy techniques: Group 1: Surgical frenectomy; Group 2: Laser frenectomy. The healing pattern of the patients was assessed using the 3 weeks post-operative healing photographs and scoring was done based on Landry, Turnbull, and Howley's healing index (1988) [15, 16 - check with author]. The data were entered into Microsoft Excel and the statistical analysis was carried out with SPSS Software, Version 23. Inferential statistics (chi-square test), as well as descriptive statistics (frequency distribution and percentage), were conducted.

## Results and Discussion:

**Figure 1:**
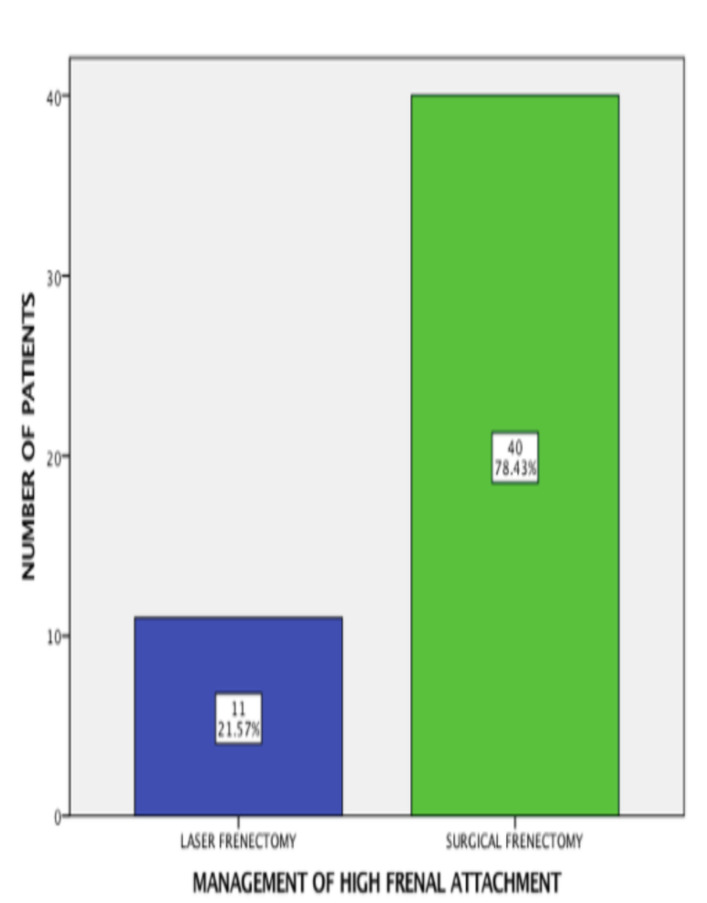
Bar chart depicting distribution of management of high frenal attachment. The X-axis illustrates management of high frenal attachment, while the Y-axis illustrates the number of patients. Most of patients had undergone surgical frenectomy (green) than laser frenectomy (blue).

**Figure 2:**
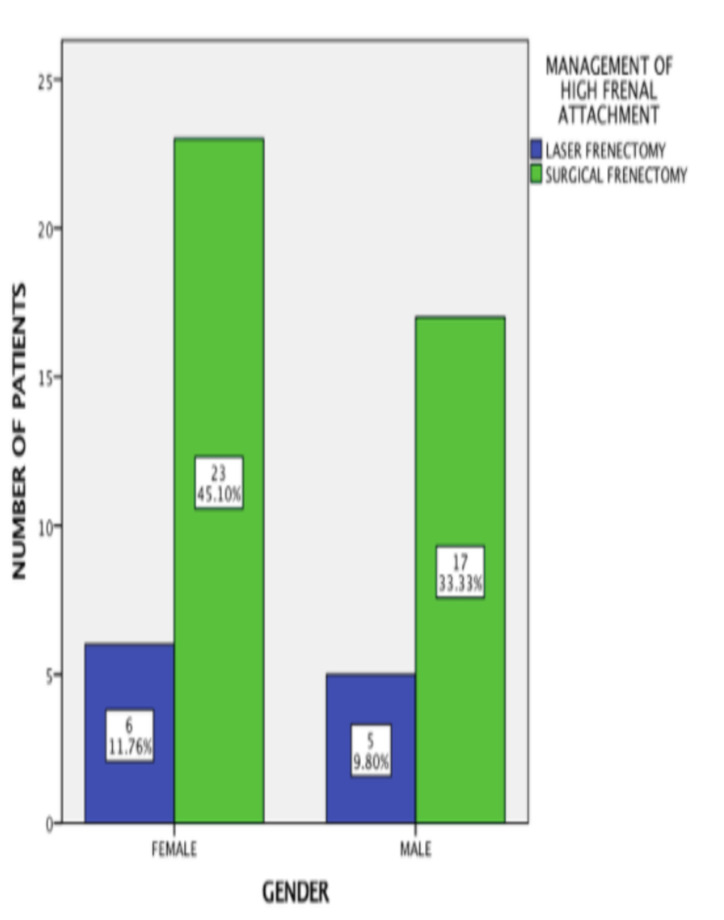
Bar chart depicting association of management of high frenal attachment based on gender. The X-axis illustrates the gender, while the Y-axis illustrates the number of patients who had undergone frenectomy. Most of the males and females had undergone surgical frenectomy (green) in comparison to laser frenectomy (blue). No statistically significant association between management of high frenal attachment and gender (Pearson Chi square value- 0.031; p =0.861).

**Figure 3:**
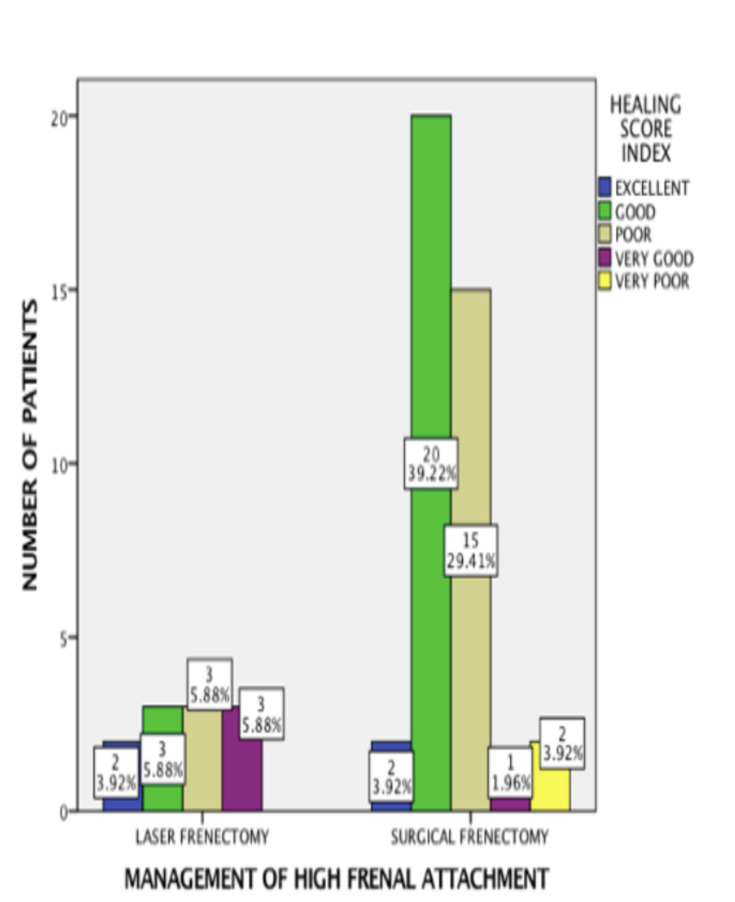
Bar chart depicting association between management of high frenal attachment and healing score index. The X-axis illustrates the high frenal attachment management, while the Y-axis illustrates the number of patients. Excellent healing score (blue) was observed between two patients in both laser and surgical frenectomy. Very poor healing score (yellow) was observed only among patients who underwent surgical frenectomy. Association between management of high frenal attachment and healing score index was statistically significant (Pearson Chi square value- 10.456; p =0.033).

**Figure 4:**
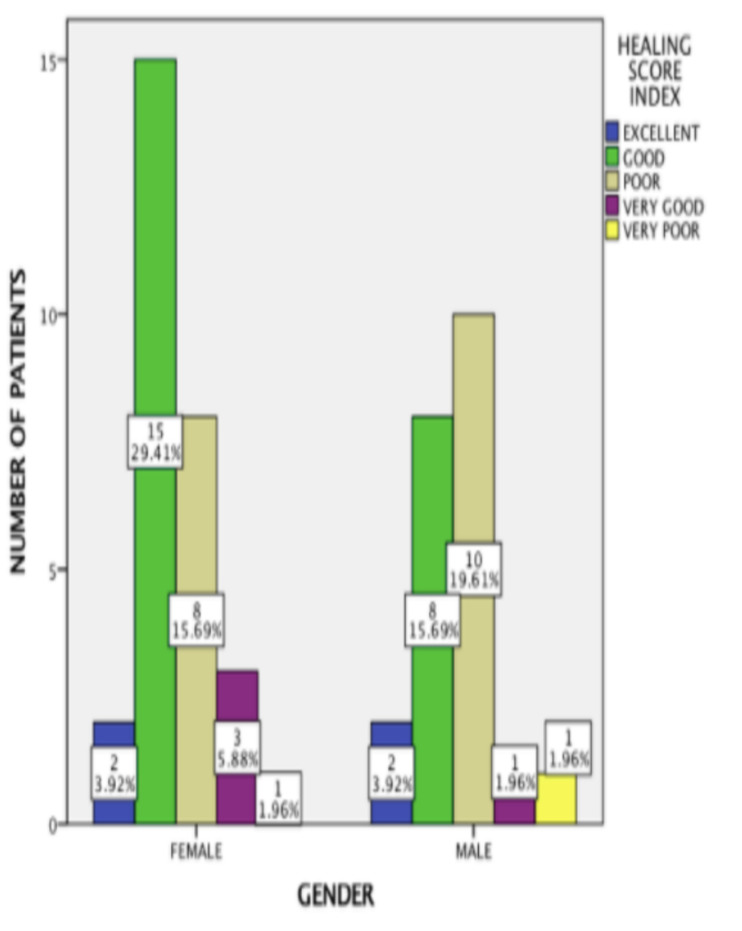
Bar chart depicting association between gender and healing score index. The X-axis illustrates the genders, while the Y-axis illustrates the number of patients based on the healing score index. Females showed the highest prevalence in good scores (green), whereas males showed the highest prevalence in poor scores (beige). Association between gender and healing score index was statistically insignificant (Pearson Chi square value- 2.438; p =0.656).

A total of 51 frenectomy patients were included in this study. Among 51 patients, 40 patients had undergone surgical frenectomy (78.43%) and 11 patients had undergone laser frenectomy (21.57%) (Figure 1). The current study participants' ages were in a range of 11 to 60 years old, with a mean age of 29.2 years. There were 29 females (56.9%) and 22 males (43.1%) among the study participants. Among 29 females, 23 patients underwent surgical frenectomy (45.1%) and 6 patients underwent laser frenectomy (11.8%). Among 22 males, 17 patients underwent surgical frenectomy (33.3%) and 5 patients underwent laser frenectomy (9.8%). The association between high frenal attachment management and gender was analyzed with the chi-square test, and it was found to be not significant statistically. (Pearson Chi-square value = 0.031; p =0.861) (Figure 2). The healing score was allotted for both methods of a frenectomy. Among 40 patients who had undergone surgical frenectomy, excellent score was observed among 2 patients (3.9%), a very good score was observed among 1 patient (2.0%), good score was observed among 20 patients (39.2%), poor score was seen among 15 patients (29.4%) and very poor score was observed among 2 patients (3.9%). Among 11 laser frenectomy patients, an excellent score was observed among 2 patients (3.9%), very good score was observed among 3 patients (5.9%), good score was observed among 3 patients (5.9%) and poor score was observed among 3 patients (5.9%). Furthermore, the chi-square test revealed a statistically significant association between the management of high frenal attachment and healing score, with a p-value of 0.033 (Figure 3). The healing index score based on gender was assessed. Among 29 females, excellent score was observed among 2 patients (3.9%), very good score was observed among 3 patients (5.9%), good score was observed among 15 patients (29.4%), poor score was observed among 8 patients (15.7%) and very poor score was observed among 1 patient (2.0%). Among 22 males, excellent score was observed among 2 patients (3.9%), a very good score was observed among 1 patient (2.0%), good score was observed among 8 patients (15.7%), poor score was observed among 10 patients (19.6%) and the very poor score was observed among 1 patient (2.0%). The chi-square test has been utilized to analyze the association between gender and the healing score index, which was considered to be insignificant with a p-value of 0.656 (Figure 4).

The majority of patients underwent conventional scalpel technique the most compared to the laser technique. Thus, the greatest prevalence was seen in surgical frenectomy. Unfortunately, no previous studies reported on the prevalence between the surgical and laser frenectomy, due to similar sample sizes used. In this study, patients preferred scalpel frenectomy because it is more economical and affordable for them [2]. It is also regarded as the gold standard technique because it requires a shorter time and is simpler to do than laser techniques [17,18]. However, the drawback of using scalpel techniques is that more intraoperative bleeding can be seen [19] along with increased pain perception and wide surgical wound and suturing [1]. This may result in postoperative discomfort for the patients.

Although conventional surgical techniques were commonly used, the laser technique was still considered the best alternative method in treating high frenal attachment. The lay public considers laser technology considered cutting-edge, with growing evidence that it creates positive therapy for periodontal disease [19]. The use of lasers during surgery will result in significantly more accurate surgical incisions and haemostasis control [20]. It is also a sutureless technique that requires less local anaesthesia and it is usually asymptomatic during recovery due to the decontaminating as well as the photobiomodulation properties of lasers [21] it is also safe to be used in all age groups including children. Operators also required both theoretical and practical training for handling lasers. During operation, some fumes are released during incisions due to the vaporization of the epithelium. Thus, it should be operated with an air evacuator [2]. Moreover, depending upon the technique used as well as the wavelength of the laser the results will be different [22].

Both techniques showed significant improvement in healing. However, laser frenectomy provided a better healing outcome than surgical frenectomy. There was no significant correlation seen between the management of high frenal attachment and the healing score index. Yadav RK et al. and Uvarshi S et al. also reported no significant difference in healing outcome using both laser and surgical techniques [3,4]. Madhuri K et al and Purushottam S et al also stated that significant improvement seen in healing with better outcome was seen in laser technique [2,15]. However, results reported by Patel RM et al were contradictory with our study. He stated that early wound healing was better seen in the conventional scalpel technique [5].

This can be explained by the primary closure of scalpel surgery, which leads to better healing during early postoperative days, while delayed healing was observed in laser surgery due to the charring and carbonization generated by laser radiation [18]. However, some claimed that lasers allowed wounds to recover quicker and create minimal scar tissue than the traditional scalpel technique [5,18,19]. Less scar tissue was seen in the laser due to coagulation of protein forming bandage over the wound area [15], which in turn creates an impermeable membrane or dressing to reduce the risk of tissue irritations and promote wound healing with less scar formation [15]. In addition to this, a greater incidence of edema and swelling may be seen in the scalpel technique due to longitudinal incisions made during surgery. However, minimal swelling and scarring would be seen in lasers due to healing by secondary intentions. This is also associated with tissue regenerations for new tissue formation on the involved areas [23-25]. Therefore, the laser technique provides better healing in comparison to the surgical techniques. Basically, the primary goal of surgical therapy for the management of high frenal attachment should demonstrate satisfactory results with minimal or no complications during or after the procedures. Thus, this will help to increase the patient's acceptance. Limitations of the study were small sample sizes with uneven numbers on distributions of patients. Hence, additional interventional research with bigger sample size is necessary to obtain precise results [26,27,28]. Further studies of frenectomy technique should incorporate different parameters such as the bleeding index and pain perceptions in order to assess the efficacy of the laser technique over the conventional surgical approaches. In the future, various techniques, as well as the long-term follow-up, should be developed to evaluate the treatment modality's stability.

## Conclusion:

Data shows that the laser technique provides better healing outcomes than the surgical technique. Moreover, the association between the management of high frenal attachment and the healing score index was found to be statistically significant.

## Author's contribution:

Nurul Afiqah Amani carried out the analysis, interpretation and drafted the manuscript. Arvina Rajasekar contributed to the conception, data design, analysis, interpretations, and critical revision of the manuscript. Shanta Sundari was involved in the research and helped to revise the paper. Each of the three authors contributed equally to each section of the manuscript.

## References

[1] Patel RM (2015). Journal of Dental Lasers..

[2] Uvarshi SRMS (2018). International Journal of Recent Scientific Research..

[3] Yadav R (2019). Journal of Indian Society of Periodontology.

[4] Singh P, Nath S (2019). International Journal of Applied DentalSciences..

[5] Huang WJ, Creath CJ (1995). Pediatr Dent..

[6] Kavarthapu A, Thamaraiselvan M (2018). Indian J Dent Res..

[7] Ramesh A (2017). J Indian Soc Periodontol..

[8] Ramamurthy J, Mg V (2018). Asian Journal of Pharmaceutical and Clinical Research..

[9] Ravi S (2017). Journal of Periodontology.

[10] Thamaraiselvan M (2015). J Indian Soc Periodontol..

[11] Arvina R, Vinoth Kumar B (2019). Research Journal of Pharmacy and Technology..

[12] Khalid W (2016). Indian Journal of Dental Research..

[13] Mootha A (2016). International Journal of Inflammation..

[14] Panda S (2014). Contemp Clin Dent..

[15] Madhuri K, Rekha B (2018). International Journal of Scientific Research..

[17] Priyanka S (2017). J Indian Soc Periodontol..

[18] Kumar R (2015). Journal Of Clinical And Diagnostic Research..

[19] Gandhi D, Gandhi P (2017). British Journal of Medicine and Medical Research..

[20] Olivi G (2009). Eur J Paediatr Dent..

[21] Olivi M (2018). Eur J Paediatr Dent..

[22] Ishikawa I (2008). J Int Acad Periodontol..

[23] Avinash K (2017). Int J Stem Cells..

[24] Varghese SS (2015). Contemp Clin Dent..

[25] Ramesh A (2016). J Intercult Ethnopharmacol..

[26] Khalid W (2017). Journal Of Clinical And Diagnostic Research..

[27] Ramesh A (2019). Journal of Indian Society of Periodontology..

[28] Ramesh A (2016). Journal of Oral Biosciences..

